# Isolation and Characterization of Pluripotent Human Spermatogonial Stem Cell-Derived Cells

**DOI:** 10.1634/stemcells.2008-0439

**Published:** 2009-01

**Authors:** Nina Kossack, Juanito Meneses, Shai Shefi, Ha Nam Nguyen, Shawn Chavez, Cory Nicholas, Joerg Gromoll, Paul J Turek, Renee A Reijo-Pera

**Affiliations:** a Institute for Stem Cell Biology and Regenerative Medicine, Department of Obstetrics and Gynecology, Stanford University School of MedicinePalo Alto, California, USA; b Center of Reproductive Medicine and Andrology, University of MuensterMuenster, Germany; c Center for Reproductive Sciences, University of CaliforniaSan Francisco, San Francisco, California, USA; d Department of Urology, University of CaliforniaSan Francisco, San Francisco, California, USA; e Sheba Medical Center, Tel HashomerIsrael

**Keywords:** Human embryonic stem cells, Germline stem cells, Adult stem cells, Spermatogonia, Testis biopsy

## Abstract

Several reports have documented the derivation of pluripotent cells (multipotent germline stem cells) from spermatogonial stem cells obtained from the adult mouse testis. These spermatogonia-derived stem cells express embryonic stem cell markers and differentiate to the three primary germ layers, as well as the germline. Data indicate that derivation may involve reprogramming of endogenous spermatogonia in culture. Here, we report the derivation of human multipotent germline stem cells (hMGSCs) from a testis biopsy. The cells express distinct markers of pluripotency, form embryoid bodies that contain derivatives of all three germ layers, maintain a normal XY karyotype, are hypomethylated at the *H19* locus, and express high levels of telomerase. Teratoma assays indicate the presence of human cells 8 weeks post-transplantation but limited teratoma formation. Thus, these data suggest the potential to derive pluripotent cells from human testis biopsies but indicate a need for novel strategies to optimize hMGSC culture conditions and reprogramming.

## INTRODUCTION

Pluripotent stem cells are characterized by their ability to proliferate and self-renew extensively, as well as to differentiate [1–6]. In humans, both human embryonic stem cell (hESC) and human embryonic germ cell (hEGC) lines were first derived in the late 1990s [7–10]. Like other pluripotent stem cells, hESCs and hEGCs are characterized by their ability to differentiate to the primary germ layers in vitro and in vivo, by expression of diagnostic cell surface markers, by specific epigenetic and genetic status, and by unique culture requirements [11, 12].

Early in fetal development, just prior to or during gastrulation, mouse germ cells are identified as a cluster of cells that are termed the primordial germ cells (PGCs) and that reside in the extraembryonic tissues at the base of the allantois [13]. These PGCs express specific mRNA and protein markers, such as tissue nonspecific alkaline phosphatase (TNAP), Oct4, and Stella, markers that are also expressed in embryonic stem cells [14–17]. Following establishment of the initial germ cell population, the PGCs expand in number and eventually migrate from the extraembryonic spaces into the nascent embryonic gonads, where they are termed gonocytes [13]. In the female, all of the immature germ cells or gonocytes enter meiosis during fetal development [18–20]. In contrast, in the male, the immature germ cells migrate to the basement membrane of the seminiferous tubules, where they differentiate into spermatogonial stem cells, which will provide the cells for differentiation of sperm throughout male adult life [21].

Over the years, culture conditions for mouse spermatogonial stem cells have been established, facilitating the characterization of these cells and factors involved in self-renewal and differentiation [22]. Nonetheless, several lines of evidence have suggested that the ability to derive pluripotent germ cell lines was restricted to the earliest stages of development (to PGCs) and that pluripotency of germ cells was not maintained postnatally [13, 18, 23–27]. However, recent results from mice have challenged this assumption [28–30]. The pluripotency of mouse spermatogonia-derived stem cells termed multipotent germline stem cells (MGSCs), multipotent adult germline stem cells, or multipotent adult spermatogonia-derived stem cells has been demonstrated by several criteria, including the ability to spontaneously differentiate into derivatives of the three primary germ layers and to contribute to chimeras [28–30]. Notably, elegant studies in mice have resulted in the identification of the progenitor population and delineation of the time course of acquisition of pluripotency. These studies have suggested that a subpopulation of cells may be “reprogrammed” to a state of pluripotency [30]. Here, we extend these studies with analysis of the derivation of putative human multipotent germline stem cells (hMGSCs) from a testis biopsy.

## MATERIALS AND METHODS

### Patient Information

Testis biopsies are routinely obtained for the diagnosis of male fertility through the clinical practice of P.J.T. and can be used for research following informed consent. In this study, 19 patient samples were obtained, and one hMGSC line (termed [NK (Nina Kossack) tissue sample 7 (NK7)]) was generated from an individual who was diagnosed with azoospermia due to acquired reproductive tract obstruction from trauma (obstructive azoospermia). This individual donor presented with normal hormone values (5 IU/l follicle stimulating hormone, 2.9 IU/l luteinizing hormone, 408 ng/dl testosterone, and 13 ng/ml prolactin), normal karyotype in a blood sample, and no detectable Y chromosome microdeletions. Histologically, testis sections showed normal spermatogenesis (supporting information data A).

### Collection of Tissue

Approximately 30–50-mg sections of testis tissue were excised and placed into minimal essential medium α (MEM-α) (Invitrogen, Carlsbad, CA, http://www.invitrogen.com). Tissue was mechanically dissected and dissociated via a two-step enzymatic incubation process: First, tissues were incubated for 30 minutes at 37°C in MEM-α containing 10 mg/ml collagenase (Invitrogen). Spermatogenic tubules, tissues, and cells were then centrifuged (5 minutes, 1,000 rpm) and resuspended in 2 ml of Hanks' balanced salt solution (Invitrogen) containing 2.2 mg/ml DNase I (Roche Applied Science, Inc., Indianapolis, http://www.roche-applied-science.com) and 4 mg/ml trypsin (Invitrogen) and incubated for 10 minutes at 37°C. Then, 2.5 volumes of MEM-α containing 10% fetal bovine serum (FBS) (Invitrogen) was added to the cell suspension; cells were washed three times with phosphate-buffered saline (PBS) (Invitrogen) and resuspended in MEM-α supplemented with 10% FBS and 1% Penicillin-Streptomycin.

### Isolation of Spermatogonial Stem Cells

Initially, we explored several methods to isolate and propagate human spermatogonial stem cells. In one approach, we treated testicular biopsy cells as described above and simply transferred the sample in total directly to gelatin-coated tissue culture plates. In a second approach, we attempted to enrich for the spermatogonial stem cell population via magnetic-activated cell sorting (MACS) for the glial cell line-derived neurotrophic factor (GDNF) family receptor-α-1 (GFR-α1) [31]. To induce the propagation of ESC-like cells from testis biopsies, we further tested alternatives of plating testicular cells directly in hESC medium without mouse embryonic fibroblasts (MEFs), plating cells in hESC medium directly onto MEFs, and plating cells in hESC medium with subsequent transfer to MEFs after 8 days; hESC medium are as described [32].

### Transfer and Culture Conditions of Spermatogonial Stem Cell Colonies

Two days postplating, most testicular cells were attached to the growing surface, and the medium was changed. After approximately 1 week, several small colonies were observed on top of the monolayer of testicular cells. To propagate these colonies under hESC conditions, they were manually transferred onto MEFs and cultured in Knockout Dulbecco's modified Eagle's medium (DMEM) (Invitrogen) supplemented with 20% FBS, 1 mM l-glutamine (Invitrogen), 0.1 mM nonessential amino acids (Invitrogen), 0.1 mM β-mercaptoethanol (Chemicon, Billerica, MA, http://www.chemicon.com), and 10 ng/ml recombinant human basic fibroblast growth factor (bFGF) (R&D Systems Inc., Minneapolis, http://www.rndsystems.com), referred to hereafter as KSR medium. To inhibit putative stem cells from differentiating, after 5 days, cells were passaged onto ultralow-attachment dishes. Manual passaging was performed for the two initial passages and was followed by enzymatic digestion using a combination of 0.01 mg/ml collagenase (100 units/ml, 37°C, 5 minutes) (Invitrogen) and trypsin (0.25% trypsin/EDTA, 37°C, 5 minutes) for later passages. Prior to transfer onto ultralow-attachment dishes (Corning Enterprises, Corning, NY, http://www.corning.com), cells were washed three times in KSR medium. After 2 days, putative stem cells were plated onto MEFs.

### RNA Isolation and cDNA Amplification

Total RNA was extracted with the RNeasy Mini Kit (Qiagen, Valencia, CA, http://www1.qiagen.com). cDNA was generated from 100 ng of RNA isolated from hMGSCs cultured on MEFs at passages 2 and 7, from hMGSCs cultured on human testicular stromal cells, and from testicular tissue (Clontech, Mountain View, CA, http://www.clontech.com) using SuperScript III Reverse Transcriptase (Invitrogen). Subsequent polymerase chain reaction (PCR) analysis was performed with Platinum Taq DNA Polymerase (Invitrogen) using 10 ng of cDNA as template to analyze expression of the genes *OCT4, SOX-2, NANOG, STELLAR, GDF3, PUMILIO1* (*PUM1*)*, PUMILIO2* (*PUM2*), *DAZL, VASA, SCP1, SCP3, MLH1, BOULE*, and *TEKT1* with primers as shown (supporting information Table 1) and cycling as follows: 94°C for 1 minute followed by 40 cycles at 94°C, 30 seconds; 60°C, 30 seconds; and 72°C, 30 seconds.

### Immunofluorescence and Alkaline Phosphatase Staining of Undifferentiated Colonies

Alkaline phosphatase staining was accomplished via the Vector Red Alkaline Phosphatase Substrate Kit I (Vector Laboratories, Burlingame, CA, http://www.vectorlabs.com), with H9 hESCs as a positive control. For immunofluorescence, undifferentiated cells were cultured on MEFs and were fixed in 4% paraformaldehyde (PFA) in PBS (pH 7.4) for 20 minutes. Cells were washed twice with PBS/0.1% Tween-20 to remove residual fixative and incubated in 1% Triton X in PBS for 30 minutes, prior to blocking in 4% normal goat serum in PBS (Jackson Immunoresearch Laboratories, West Grove, PA, http://www.jacksonimmuno.com) for 30 minutes followed by incubation with antibody solution overnight at 4°C. Primary antibodies included: OCT4 (1:200; Santa Cruz Biotechnology Inc., Santa Cruz, CA, http://www.scbt.com), SOX2 (1:200; Chemicon), stage-specific embryonic antigen 4 (SSEA4) (1:200; Chemicon), TRA1–81 (1:200; Chemicon), and DAZL (1:100, protocol as described [32]). The following day, cells were washed twice with PBS/0.1% Tween-20, 5 minutes, and incubated with appropriate secondary antibody (1:200; Invitrogen) in PBS. After two washes with PBS + 0.1% Tween-20 for 5 minutes, cells were mounted with anti-fade mounting media or 4,6-diamidino-2-phenylindole (DAPI)/PBS and viewed on a Leica DM IL microscope (Leica, Heerbrugg, Switzerland, http://www.leica.com) or on a Zeiss LSM 510 Confocal Laser Scanning Microscope (Carl Zeiss, Jena, Germany, http://www.zeiss.com) equipped for two-photon excitation.

### Spectral Karyotyping

Growing colonies were incubated with 0.1 μg/ml colcemid (Gibco, Grand Island, NY, http://www.invitrogen.com) at 37°C overnight. Cells were enzymatically detached as described above and resuspended in KSR medium. To achieve single-cell suspension, cells were pelleted at 1,000 rpm for 5 minutes, resuspended in 0.25% trypsin/EDTA (Gibco), and incubated at 37°C for 5 minutes. KSR was added to inactivate the trypsin; cells were pelleted and resuspended in 0.4% sodium citrate and 0.4% KCl at a 1:1 ratio and incubated at 37°C for 15 minutes. An equal volume of Carnoy's solution (3:1 ratio of methanol to acetic acid) was added, followed by incubation at room temperature for 5 minutes, to fix cells (this step was repeated twice with fresh fixative). Finally, pellets were resuspended in a small volume of fixative and transferred to microscope slides. Spectral karyotyping (SKY) analysis was performed using SkyPaint Human H-10 according to the manufacturer's instructions (Applied Spectral Imaging, Vista, CA, http://www.spectral-imaging.com) and visualized on a Leica DMR Microscope with an Applied Spectral Imaging SD-301-VDS unit.

### Short Tandem Repeat/Variable Number of Tandem Repeat Analysis

Genomic DNA was extracted from hMGSCs via the QIAamp DNA Mini system (Qiagen) and from tissue donor blood via the QIAamp DNA Blood Maxi Kit (Qiagen). Genomic DNA from the hESC line H9 was used as a negative control. Ten microliters of genomic DNA at a concentration of 2.5 ng/μl was submitted for analysis via AmpFeSTR Identifiler PCR amplification (Applied Biosystems, Foster City, CA, http://www.appliedbiosystems.com). Fifteen tetranucleotide repeat loci and the amelogenin gender determining marker were analyzed.

### Bisulfite Sequencing Analysis

hMGSCs were cultured in feeder-free conditions for 2 days, collected, washed with PBS, quick-frozen on dry ice, and stored at −80°C. H9 hESCs and sperm and whole-blood genomic DNA served as controls. Genomic DNA was extracted via the QIAamp DNA Mini system. Conversion of unmethylated cytosines was performed via the Methyl Easy Xceed Rapid DNA Bisulphite Modification Kit (Human Genetic Signatures, Sydney, New South Wales, Australia, http://www.geneticsignatures.com). For bisulfite treatment, 0.5–1 μg of genomic DNA was used, resulting in a final converted DNA concentration of 15–20 ng/μl. Four microliters of product was amplified. Seminested PCR was performed via two rounds: (a) 94°C, 10 minutes, followed by 30 cycles of 94°C for 45 seconds, and 61°C for 45 seconds, 72°C for 1 minute, and a final extension step of 72°C for 10 minutes; (b) 35 cycles (same conditions but second set of primers). Primers were human-specific *H19* forward, 5′-AGGTGTTTTAGTTTTATGGATGATGG-3′; *H19* forward 2, 5′-TGTATAGTATATGGGTATTTTTGGAGGTTT-3′; and *H19* reverse, 5′-TCCTATAAATATCCTATTCCCAAATAACC-3′, as described in Kerjean et al. [33]. PCR products were gel purified and cloned into a TOPO vector (Invitrogen). In addition, the DNA methylation profile of the 5′-flanking region of the human *OCT4* gene was analyzed. The region that was investigated was between −2,564 and +153 base pairs (bp) from the transcription start site and contained the proximal enhancer (PE), the distal enhancer (DE), and the proximal promoter (PP), as indicated in Figure 3. Primer pairs *OCT*4–2 forward (F)/2 reverse (R), *OCT*4-3F/3R, *OCT*4-5F/R, and *OCT*4-9F/R and PCR conditions were used as described in Deb-Rinker et al. [34].

### Telomerase Activity

Telomerase activity was analyzed in duplicates using the TRAPeze ELISA Telomerase Detection Kit (Chemicon). Cells were grown, feeder-free, for 2 days, collected, washed with PBS, and subsequently quick-frozen on dry ice. The hESC line HSF8 (XY) was used as a positive control. Cells were lysed in 1× 3-[(3-cholamidopropyl)dimethylammonio]-1-propanesulfonic acid (CHAPS) lysis buffer, and protein concentration was determined via BCA assay (Pierce, Rockford, IL, http://www.piercenet.com). Sample extracts were diluted 1:100 with 1× CHAPS lysis buffer, using approximately 3 ng of protein per extract for the TRAPeze ELISA Telomerase Detection Kit assay. Amount of product was determined using a Multiscan EX automatic microplate reader (Thermo, Inc. Milford, MA, http://www.thermo.com). Absorbance was measured at 450 and 620 nm, and telomerase activity was determined as follows: absorbance = A_450_ − A_620_. Heat-treated extracts (99°C for 20 minutes) were analyzed in parallel as a negative control.

### Differentiation of hMGSCs

To induce embryoid body (EB) formation, hMGSCs were dissociated with trypsin, neutralized with KSR medium, and washed three times with differentiation medium (Knockout DMEM supplemented with 20% FBS, 1 mM l-glutamine, 0.1 mM nonessential amino acids, and 0.1 mM β-mercaptoethanol) prior to transfer onto ultralow-attachment dishes. One-third of the resulting EB suspension was collected on days 0, 3, 7, 11, 14, and 21 to determine differentiation status at these time points. RNA was isolated via the Pico Pure RNA isolation kit (Arcturus, Mountain View, CA, http://www.arctur.com), transcribed into cDNA via the WT-Ovation RNA Amplification System (NuGEN, San Carlos, CA, http://www.nugeninc.com), and analyzed. H9 hESCs were used as a positive control for each differentiation experiment. Real-time PCR using TaqMan Gene Expression Assays (Applied Biosystems) was performed to determine the expression levels of *OCT4* (Hs01895061_u1), *MSI1* (Hs 00159291_m1), *GATA4* (Hs 00171403_m1), and neural cell adhesion molecule (NCAM) (Hs00169851_m1) using 20 ng of cDNA per reaction. Expression values were normalized for *GAPDH* and calculated as previously described [35]. *KDR* expression was analyzed using SYBR green (Applied Biosystems) reverse transcriptase (RT)-PCR. All experiments included controls without any cDNA template for each primer set.

For teratoma assays, cells were cultured under feeder-free conditions for 2 days, incubated in 0.25% trypsin/EDTA for 5 minutes at 37°C, and transferred to KSR medium supplemented with 20% FBS and 10 ng/μl bFGF. After two washes, cells were resuspended in 1 ml of KSR and aliquoted into two 0.5-ml tubes. Cell pellets were collected to prepare two grafts. Phytohemagglutinin was added to a final concentration of 0.2 mg/ml, cells were pelleted by centrifugation at 10,000*g* for 1 minute, and the cell pellet was incubated for 5 minutes at room temperature. The two cell pellets were transferred into 0.4-μm Millicell-CM inserts (Millipore, Temecula, CA, http://www.millipore.com) in a 2-cm dish containing KSR medium. Grafts were incubated overnight at 37°C and implanted under the kidney capsule of a female SCID recipient mouse, as described at http://mammary.nih.gov/tools/mousework/Cunha001. Grafts were harvested and fixed with 4% PFA 8 weeks post-transplantation. Fixed tissue was paraffin embedded, sectioned, and stained with hematoxylin and eosin.

To investigate whether teratoma formation would be more efficient if larger cell numbers and/or support cells were transplanted, approximately 10,000 hMGSCs were combined with 1 million irradiated MEFs. After two washes, cells were resuspended in 1 ml of KSR medium and aliquoted into two 0.5-ml tubes. Cell pellets were collected to prepare two grafts, and transplantation of the grafts was performed as described above. One million irradiated MEF cells were used to prepare two grafts, which served as a negative control.

To analyze the origin of cells in grafts, genomic DNA was isolated using the QIAamp DNA Mini system (Qiagen). Sixty nanograms of DNA was used as a template to detect human *sex determining region Y* (*SRY*). A 350-bp fragment was amplified using primers *SRY* forward, 5′-CGCATTCATCGTGTGGTCTCG-3′, and *SRY* reverse, 5′-AGCTGGTGCTCCATTCTTGAG-3′. PCR was performed as follows: 94°C for 1 minute and 35 cycles of 94°C for 1 minute, 58°C for 45 seconds, and 72°C for 45 seconds. Resulting DNA fragments were separated by gel electrophoresis. Samples included NK7 hMGSC grafts, NK7 hMGSC genomic DNA, human sperm genomic DNA, and genomic DNA isolated from the tail tip of a female SCID mouse.

### Immunofluorescence Staining of Differentiated hMGSCs

To assess differentiation, hMGSCs were differentiated in EBs for 7 days; then, EBs were plated onto gelatin-coated dishes approximately 12 hours prior to immunofluorescence analysis. Markers used were the endoderm marker von Willebrand factor (VWF), the mesoderm marker α-smooth muscle actin (ASMA), and the ectoderm marker nestin (NES). Prior to staining, cells were fixed with 4% PFA for 15 minutes, fixed cells were then washed with PBS and blocked in PBS-BT for 30 minutes. Cells were incubated for 90 minutes with primary antibodies for VWF (1:400; Abcam, Cambridge, MA, http://www.abcam.com) and ASMA (1:20, Abcam) and overnight at 4°C with the NES primary antibody (1:100; Abcam). Following incubation, cells were washed with 0.3% bovine serum albumin (Sigma-Aldrich, St. Louis, http://www.sigmaaldrich.com) plus 0.1% Triton X-100 (Sigma-Aldrich) in PBS (PBS-BT) for 5 minutes, stained with the corresponding secondary antibody at a 1:200 dilution for 1 hour, washed three times in PBS-BT, counterstained with DAPI, and viewed on a Leica DM IL microscope.

### Immunofluorescence Staining Following Induced Neural Differentiation

NK7 hMGSCs were plated onto gelatin and were cultured until 80% confluence was achieved. Subsequently, KSR medium was replaced with DMEM/F12 + GlutaMAX medium (Invitrogen) supplemented with N-2 (Invitrogen) for 2 weeks. After 2 weeks in culture, the medium was then changed to Neurobasal Medium (Invitrogen) supplemented with B-27 (Invitrogen) for 4 weeks. Subsequently, the expression of ectodermal-specific markers, such as NES, microtubule-associated protein 2 (MAP2), and β-tubulin III (TUB III), was analyzed by immunofluorescence staining. NES primary antibody was used at a dilution of 1:100 (Abcam), MAP2 (Chemicon) at 1:200, and TUB III (Covance, Berkeley, CA, http://www.covance.com) at 1:750.

## RESULTS

### Isolation of hMGSCs from Human Testis Biopsies

In initial attempts to isolate hMGSCs, we obtained testis biopsies and generated cell suspensions by enzymatic digestion. We then sought to enrich for the spermatogonial stem cell population by MACS with the cell surface marker GFR-α (the receptor for GDNF). GFR-α had previously been reported to localize to a subset of type A spermatogonia in mice [36]. Isolated cells were cultured on gelatin-coated dishes in MEM-α. However, although the resulting cells were capable of being propagated in vitro, they had an elongated spindle-shaped appearance (similar to fibroblasts), distinctly different from that of hESCs, and lacked characteristic expression of cell surface markers of pluripotent cells.

Thus, we explored alternative methods to induce the propagation of hESC-like cells from testis biopsies: (a) culture of testicular cells in hESC medium post-biopsy digestion; (b) culture of testicular cells in hESC medium for 8 days postdigestion, with subsequent transfer onto MEFs; and (c) transfer directly onto MEFs in hESC media. We noted that all three of these approaches, in contrast to MACS separation, resulted in the formation of colonies. However, these colonies could not be successfully propagated in vitro; with passaging via trypsin digestion, the cultures would progressively become devoid of stem cell-like cell colonies. Thus, in 17 of 17 biopsies subjected to these protocols, no hMGSC line was derived. In contrast, as described below, by manual passaging we succeeded in the derivation of two hMGSC lines (although one patient withdrew from the study, and materials were discarded in that case).

**Figure 1:**
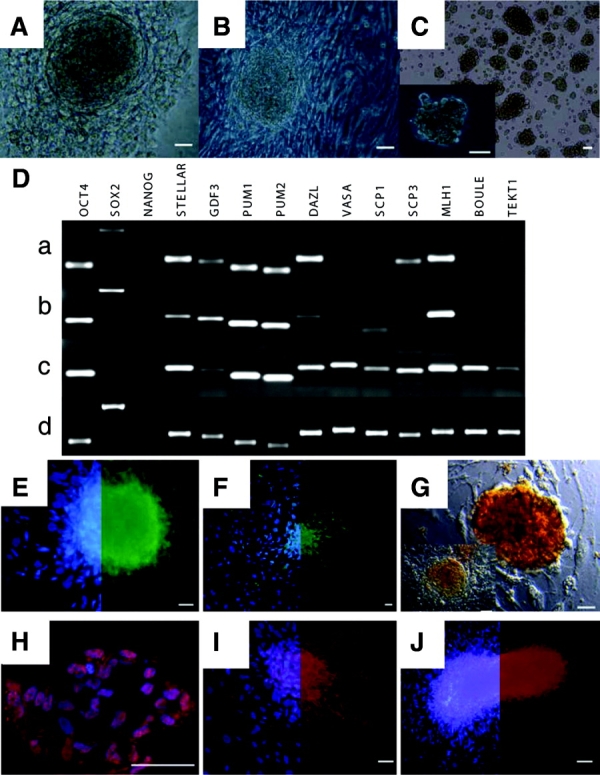
Morphological analysis and analysis for the expression of both pluripotency- and germ cell-specific markers. **(A):** Light microscopy of spermatogonial stem cell colonies growing on top of a monolayer of testicular cells approximately 2 weeks after plating of the testicular cell suspension. **(B):** After six passages in culture. **(C):** Spermatogonial stem cells in suspension. **(Da):** Expression analysis of embryonic stem cell- and germ cell-specific markers in NK (Nina Kossack) tissue sample 7 (NK7) human multipotent germline stem cells (hMGSCs) cultured on mouse embryonic fibroblasts after two passages and seven passages. **(Db, Dc):** Expression analysis of NK7 cells cultured on human testicular stromal cells after 10 passages and of a commercially available testis sample. **(Dd):** Reverse transcriptase-polymerase chain reaction products separated by gel electrophoresis. **(E–H):** Immunofluorescence staining of hMGSCs at passage 8, for pluripotency markers: **(E):** stage-specific embryonic antigen 4. **(F):** TRA1–81. **(G):** Staining of hMGSCs at passage 8 for embryonic stem cell and germ cell-specific marker alkaline phosphatase. Shown are two colonies with different morphologies. **(H):** OCT4. **(I):** SOX2. **(J):** Staining of hMGSCs at passage 8 for embryonic stem cell and germ cell-specific marker DAZL. **(E, F, I, J)** show the colocalization of 4,6-diamidino-2-phenylindole with the corresponding pluripotency-germ cell marker on the left side and the respective marker on the right side of the image. Scale bars = 50 μm.

As an alternative, manual passaging of colonies was explored. Following enzymatic dissociation of the testis biopsy, after approximately 7–10 days of culture, very small colonies started to grow on top of the monolayer of testicular cells; these colonies were manually transferred onto MEFs and cultured under hESC conditions (Fig. 1 A, 1B). These cells, which we have termed hMGSCs, have been propagated for approximately 20 passages in vitro; the current line is designated NK7. The putative NK7 hMGSCs were passaged once every week and have maintained the ability to form colonies with characteristic hESC morphology. However, although the cells in the middle of the colonies have a distinctive hESC-like appearance, some of the cells at the periphery appear to differentiate and acquire a spindle-shaped morphology, suggesting the need to optimize medium and/or culture and derivation conditions (Fig. 1B). In suspension, NK7 cells continued to divide and formed EB-like structures (Fig. 1C).

### Gene Expression Analysis

RT-PCR was performed to analyze the expression of a subset of pluripotency markers, as well as germ cell-specific genes, in the isolated hMGSCs at passages 2 and 7 relative to a normal human testis sample (Fig. 1D). Results demonstrated that the hMGSCs at passages 2 and 7, grown on MEFs, express a subset of those genes expressed in the testis, as shown (compare Fig. 1 Da, 1 Db with Fig. 1 Dd), which includes the pluripotency markers *OCT4* (octamer-binding transcription factor-4) and *SOX2* (SRY-box 2). *NANOG* expression, however, could not be detected in either the isolated hMGSCs or the testis sample. Apart from that, expression of the hESC- and germ cell-enriched genes *STELLAR* (*STELLA-related*), *GDF3* (*growth and differentiation factor 3*), *PUM1* (*PUMILIO 1*), and *PUM2 (PUMILIO 2*) was observed. In addition, the hMGSCs expressed the germ cell-specific gene *DAZL* (*Deleted in AZoospermia-Like*), as well as *SCP3* (*Syntaptonemal Complex Protein 3*) and *MLH1* (*Mut-L Homolog 1*). In contrast, expression of the markers *VASA* and *SCP1* was not detected, nor was the expression of the two developmentally late germ cell markers *BOULE* and *TEKT1* (Fig. 1D). Notably, however, when we cultured NK7 cells on human testicular stromal cells, we observed the induction of expression of later germ cell markers, including *BOULE* and *TEKT1*, and loss of *SOX2* expression (Fig. 1 Dc). We therefore concluded that NK7 cells lose the expression of later germ cell markers if cultured under human ESC conditions and regain the expression of pluripotency genes, such as *SOX2*, if cultured on MEFs in human ESC conditions.

Our next aim was to examine the expression of pluripotency markers in hMGSCs by immunofluorescence (Fig. 1E–J). Putative hMGSCs were shown to express the human pluripotency markers SSEA4 (Fig. 1E), TRA1–81 (keratin sulfate-related antigens; Fig. 1F), OCT4 (Fig. 1H), and SOX2 (Fig. 1I). In addition, the hMGSCs also stained positive for the early germ cell and hESC marker TNAP (Fig. 1G), as well as the germ cell lineage marker DAZL (Fig. 1J). Negative controls for all experiments demonstrated that antibodies were specific, as expected.

### Spectral Karyotype

**Figure 2:**
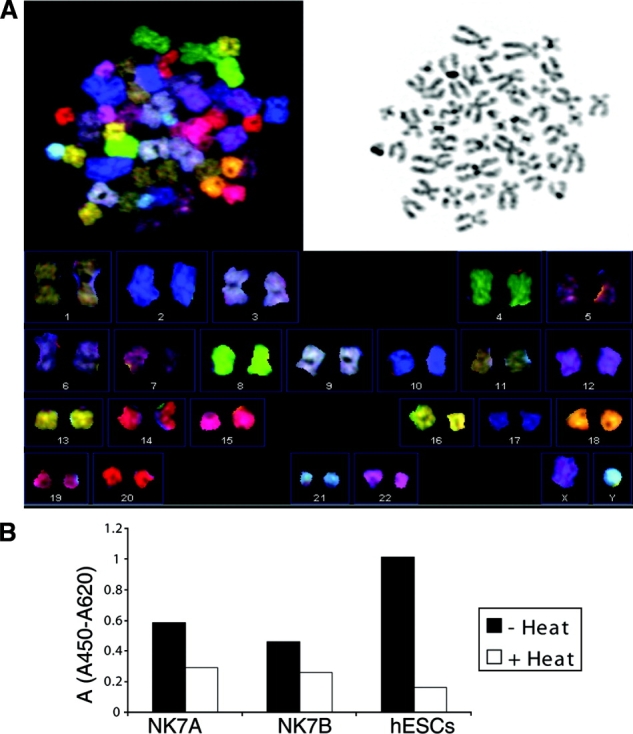
Human multipotent germline stem cells (hMGSCs) have a normal karyotype and express telomerase. **(A):** The DNA spectral karyotyping experiment of undifferentiated hMGSCs at passage 8 demonstrates a normal (46,XY) karyotype. The spectral image, the 4,6-diamidino-2-phenylindole staining, and the resulting chromosome table are shown. **(B):** Telomerase activity was investigated in hMGSCs at passage 6 (NK7A) and passage 8 (NK7B) using the TRAPeze ELISA. A cell extract from HSF8 hESCs served as a positive control for this experiment. The telomerase activity was calculated as average change in absorbance of the sample at 450 nm minus the absorbance at 690 nm. The black bars represent the samples without heat treatment, and the white bars represent the samples with heat treatment. Abbreviations: A, absorbance; hESC, human embryonic stem cell.

To determine the karyotype of the derived NK7 hMGSC line, SKY analysis was performed. Results demonstrated that the NK7 hMGSC line has a normal karyotype (46, XY) and no Y chromosome microdeletions. No indications of other cytogenetic abnormalities were detected (Fig. 2A). This indicated that the derived cell line was karyotypically identical to the patient's somatic cells, at this level of analysis.

### Telomerase Activity and Methylation of the H19 Differentially Methylated Region and the OCT4 Promoter Region

Telomerase activity is indicative of pluripotent stem cells. We examined telomerase activity of the hMGSCs at passages 6 and 8 relative to the human XY-bearing ESC line HSF8, as a positive control. As expected, hESCs exhibited a very high telomerase activity with little or no residual activity in the heat-inactivated control. Telomerase activity was also detected in the two hMGSC extracts, with the level of telomerase activity slightly reduced in cells that had been cultured for eight passages relative to those cultured for six passages (Fig. 2B).

### Short Tandem Repeat/Variable Number of Tandem Repeat Analysis

Short tandem repeat (STR)/variable number of tandem repeat (VNTR) analysis was performed to determine the origin of the NK7 hMGSCs. Samples analyzed were genomic DNA isolated from NK7 hMGSCs, genomic DNA from the tissue donor's blood sample, and genomic DNA from H9 hESCs. The results (Table 1) demonstrate that the number of short tandem repeats on both alleles of the 15 loci that were analyzed is identical in NK7 hMGSCs and the tissue donor's blood sample. The probability that two randomly selected individuals would have an identical genotype at these 15 loci is minuscule (5.01 × 10^−18^ [37]). Although H9 cells have the same number of short tandem repeats as the NK7 hMGSCs on both alleles of the *HUMTHO1* locus and on one allele of the *D16S539*, *D18S51*, and *D5S818* loci, the number of short tandem repeats at all other examined loci differed between H9 hESCs and the NK7 hMGSC line.160:

**Table 1:** Variable number of tandem repeat/STR analysis of 15 STR loci

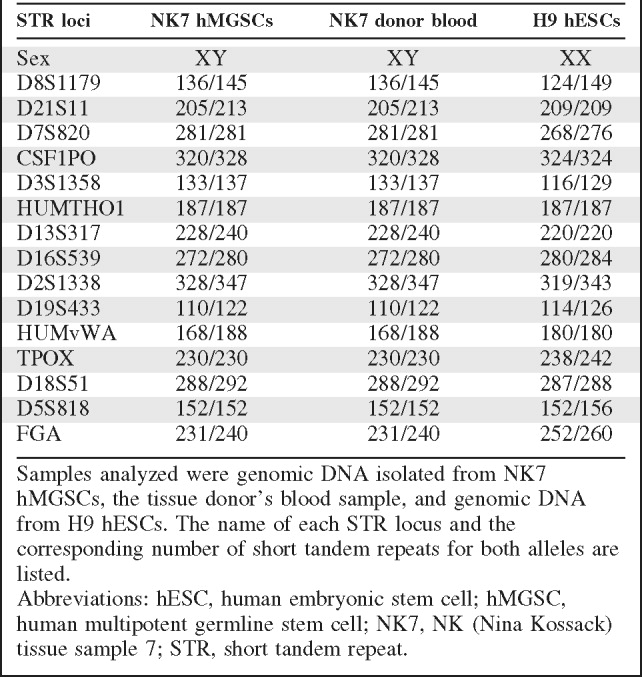

Samples analyzed were genomic DNA isolated from NK7 hMGSCs, the tissue donor's blood sample, and genomic DNA from H9 hESCs. The name of each STR locus and the corresponding number of short tandem repeats for both alleles are listed.

Abbreviations: hESC, human embryonic stem cell; hMGSC, human multipotent germline stem cell; NK7, NK (Nina Kossack) tissue sample 7; STR, short tandem repeat.

### Bisulfite Sequencing

**Figure 3:**
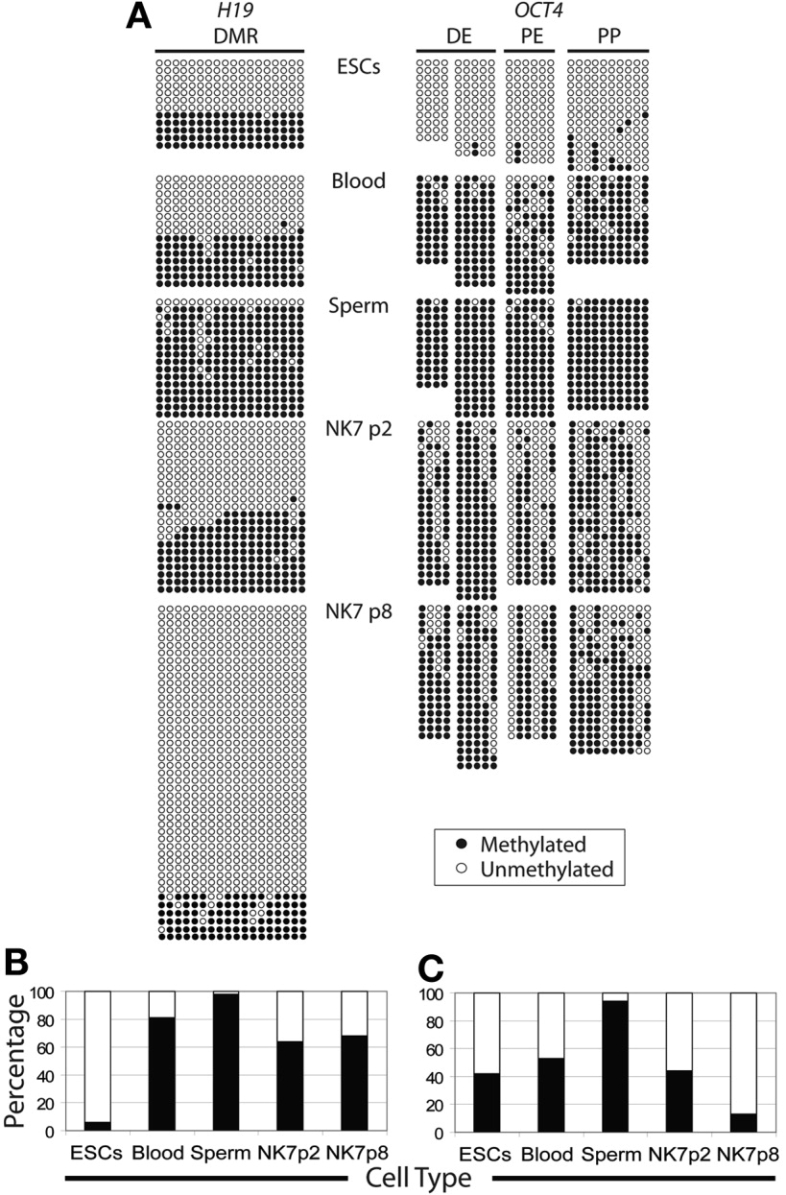
Bisulfite sequencing of the *OCT4* promoter region and the DMR, located upstream of the *H19* promoter. **(A):** Methylation profile of the human *OCT4* and *H19* genes. Each row of circles represents a single cloned allele, and each circle represents a single CpG site (white circles, nonmethylated cytosine; black circles, methylated cytosine). Bisulfite-modified DNA from H9 human embryonic stem cells, human multipotent germline stem cells (NK7) at p2 and p8, blood cells, and sperm cells were analyzed. **(B):** Percentages of methylated and unmethylated clones present in the DMR of the *H19* gene, as portrayed in **(A)** above. White bars indicate the percentages of unmethylated clones, and black bars indicate the percentages of methylated clones for each cell type as shown along the *x*-axis. **(C):** Percentages of methylated and unmethylated clones present in the promoter region of the *OCT4* gene, as portrayed in **(A)**. White bars indicate the percentages of unmethylated clones, and black bars indicate the percentages of methylated clones for each cell type as shown along the *x*-axis. Abbreviations: DE, distal enhancer; DMR, differentially methylated region; NK7, NK (Nina Kossack) tissue sample 7; p, passage; PE, proximal enhancer; PP, proximal promoter.

Bisulfite sequencing was performed to investigate the methylation status of 18 CpG (cytosine guanine) dinucleotides in the differentially methylated region upstream of the *H19* promoter (Fig. 3A). Although the maternal *H19* allele is active and therefore unmethylated, the paternal *H19* allele is methylated in all somatic cells [38, 39]. Human ESCs, as well as human somatic cells, carry one paternal and one maternal allele and showed a ratio of 70%:30% and 50%:50% unmethylated to methylated sequences, respectively, as shown (Fig. 3B). In contrast, in mature sperm, the paternal allele of the *H19* gene was completely methylated (100% of clones), indicative of the establishment of the unique male-specific methylation pattern at this locus during this stage of development, (Fig. 3A). In contrast, when we examined the methylation status of *H19* in NK7 hMGSCs at passage 8, we observed that this locus was hypomethylated, with 87% of clones unmethylated and only 13% methylated (Fig. 3 A, 3C).

In addition, the DNA methylation profile of the 5′-flanking region of the human *OCT4* gene was analyzed. The region investigated contains the PE, the DE, and the PP, as indicated in Figure 3 A. In undifferentiated cells the majority of CpG repeats in this region are unmethylated and the gene is therefore expressed. Analysis showed that 94% of CpG sites in the *OCT4* promoter region of human ESCs are unmethylated, whereas only 19% of CpG repeats in blood cells and 2% of CpG repeats in sperm cells were unmethylated. Analysis of the methylation status of the *OCT4* promoter region of NK7 cells at passages 2 and 8 showed that 36% and 32% of CpG repeats were unmethylated, respectively (Fig. 3 A, 3C). This partial demethylation is in accordance with the finding that the *OCT4* gene is activated in NK7 cells, as demonstrated by RT-PCR and immunofluorescence staining.

### Spontaneous Differentiation

**Figure 4:**
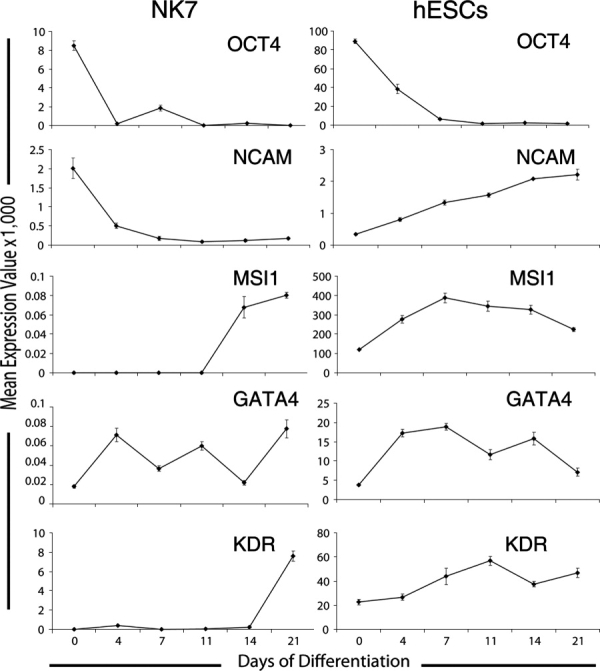
In vitro differentiation of human multipotent germline stem cells (hMGSCs). hMGSCs and H9 human embryonic stem cells were spontaneously differentiated in vitro over a period of 21 days. RNA was isolated at six time points, and TaqMan reverse transcriptase-polymerase chain reaction was performed to quantify the expression of the pluripotency marker *OCT4* and the somatic genes *NCAM*, *MLH1* (ectoderm marker), *GATA4* (endoderm marker), and *KDR* (mesoderm marker). Expression values were calculated as previously described [35] and were normalized using *GAPDH* as a reference. Abbreviation: NCAM, neural cell adhesion molecule.

Pluripotent stem cells can self-renew or differentiate to the three primary germ layers: endoderm, mesoderm, and ectoderm. To assess whether hMGSCs are able to spontaneously differentiate into derivatives of the three germ layers in vitro, expression of ectoderm-, endoderm-, and mesoderm-specific genes and proteins was analyzed at different time points during differentiation. H9 hESCs were used as a positive control (Fig. 4). As shown, expression of the pluripotency marker *OCT4* decreased with differentiation, with a concomitant increase in the expression of the somatic markers *MSI1* (ectoderm marker), *GATA4* (endoderm marker), and *KDR* (mesoderm marker) in both hMGSCs and hESCs. Notably, we also found that although *NCAM* is commonly used as an ectoderm marker in hESC research and would thus be expected to increase with differentiation, its expression decreased with differentiation of hMGSCs. This is contrast to hESCs, which exhibited an increase in the expression levels of *NCAM* (Fig. 4).

**Figure 5:**
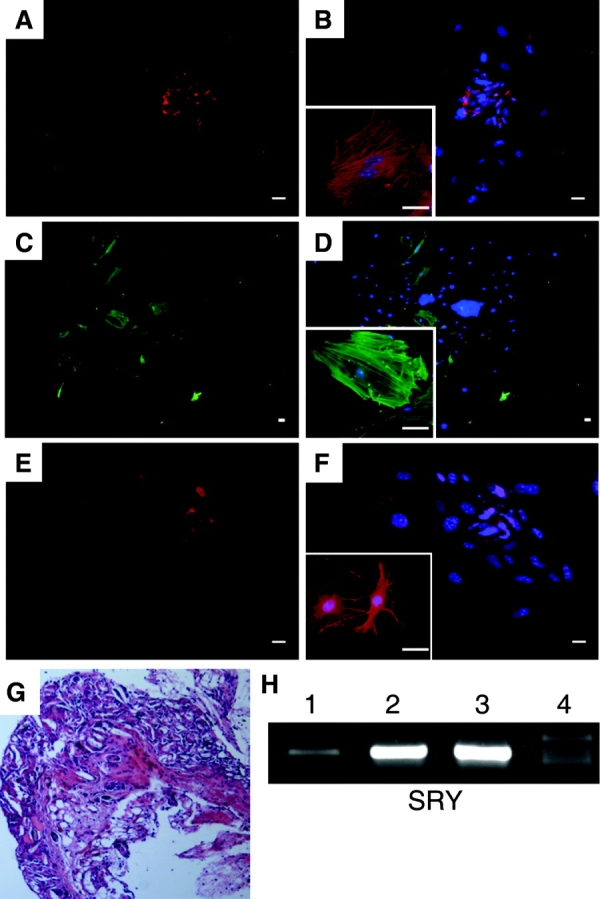
Immunofluorescence staining of day 7 differentiated human multipotent germline stem cells and teratoma analysis. Attached EBs were assessed for protein expression as shown in **(A-F)**. **(A):** von Willebrand factor (VWF). **(B):** VWF with 4,6-diamidino-2-phenylindole (DAPI) overlay. **(C):** α-Smooth muscle actin (ASMA). **(D):** ASMA with DAPI overlay. **(E):** Nestin (NES). **(F):** NES with DAPI overlay. Scale bars = 50 μm. **(G):** Analysis of teratomas 8 weeks post-transplantation. Shown is a representative section of the NK (Nina Kossack) tissue sample 7 (NK7) cell graft after 8 weeks of in vivo differentiation. **(H):** Expression analysis of the human *SRY* gene was performed using genomic DNA isolated from an NK7 paraffin-embedded tissue slide (lane 1), NK7 genomic DNA (lane 2), sperm genomic DNA (lane 3), and female mouse genomic DNA as template (lane 4). Shown are polymerase chain reaction products separated by gel electrophoreses. The fragment amplified with the SRY primers had a size of 350 base pairs. Abbreviation: *SRY*, *sex determining region Y*.

Once we examined the expression of ectoderm-, endoderm-, and mesoderm-specific genes at the mRNA level, our next aim was to evaluate germ layer marker expression at the protein level by immunofluorescence. After 7 days of in vitro differentiation, differentiated hMGSCs were positive for the endoderm-specific VWF (Fig. 5 A, 5B); ASMA, which specifically recognizes α-smooth muscle actin (Fig. 5 C, 5 D; mesoderm); and NES, an intermediate filament that is expressed in early embryonic neuroepithelial stem cells (Fig. 5 E, 5 F; ectoderm).

Finally, we tested the ability of hMGSCs to form teratomas under the kidney capsule of a female immunodeficient (SCID) mouse to investigate their differentiation capacity in vivo. The grafts were recovered 2 months post-transplantation and weighed 1.2 and 0.5 mg; histological evaluation showed that extensive teratoma formation was not detected (Fig. 5G). However, as discussed further below, human cells were present in the graft after 2 months, as demonstrated by molecular analysis (Fig. 5H). PCR analysis of the human *SRY* gene product indicated that the two positive control samples, NK7 genomic DNA and sperm genomic DNA, contained a specific 350-bp band, as did the NK7 hMGSC graft DNA. In contrast, no specific band was amplified using female mouse genomic DNA as a template. To investigate whether teratoma formation is supported by an increased cell number and/or support cells, grafts were prepared using approximately 10,000 hMGSCs accompanied by 1 million irradiated MEFs as carrier cells. Again, the grafts were recovered 2 months post-transplantation; histological analysis again revealed a variety of cell types present but no wide-scale expansion to large teratomas as is frequently seen with hESCs.

### Immunofluorescence Staining Following Induced Neural Differentiation

**Figure 6:**
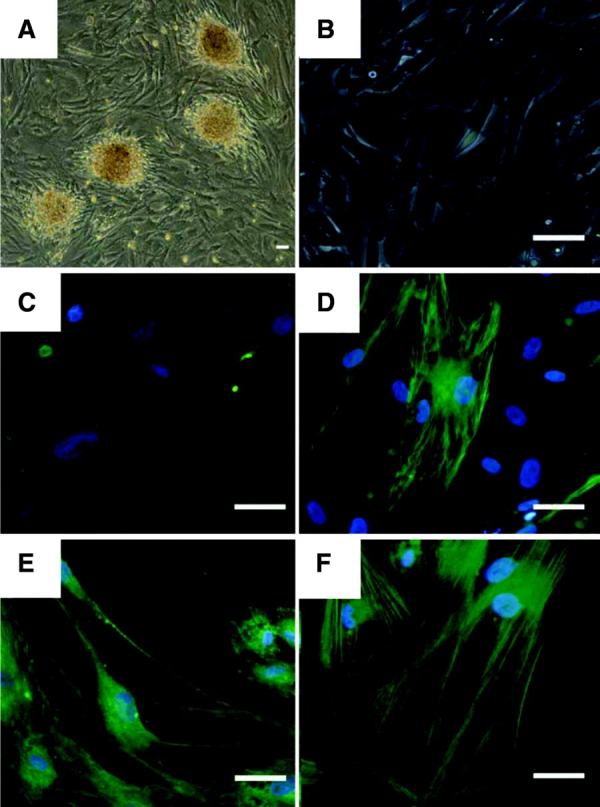
Immunofluorescence staining after 6 weeks of induced neural differentiation. Human multipotent germline stem cells were cultured on mouse embryonic fibroblasts in human embryonic stem cell media **(A)** and were plated onto gelatin **(B)** prior to the differentiation experiment. Cells were stained for nestin **(C)** before the treatment and following induced differentiation **(D)**. Differentiated cells were also stained for the ectodermal markers microtubule-associated protein 2 **(E)** and β-tubulin III **(F)**. Scale bars = 50 μm.

NK7 hMGSCs formed colonies when they were cultured on MEFs (Fig. 6 A), whereas they grew as a monolayer when they were cultured on gelatin (Fig. 6B). Prior to differentiation, the hMGSCs were plated onto gelatin and were cultured until 80% confluence was achieved. Subsequently, with 6 weeks of induced differentiation to the neural cell lineage, immunofluorescence staining of neural makers was performed on the NK7 line. Cells positive for NES could be detected after the induced differentiation (Fig. 6D) but not in the untreated cell population (Fig. 6C). In addition, cells stained positive for MAP2 (Fig. 6E) and TUB III (Fig. 6F), demonstrating that NK7 hMGSCs have the potential to differentiate toward the ectodermal (neural) lineage.

## DISCUSSION

Standard methods of generating hESC lines are limited in their ability to generate patient- or disease-specific lines of potential use for both basic science and clinical applications. However, over the years, elegant studies have shown that in model organisms and/or humans, somatic cells can be reprogrammed to an undifferentiated state via methods such as somatic cell nuclear transfer, somatic cell fusion with embryonic stem cells, and induced pluripotent stem cell technology [40–43]. In addition, recent reports have documented the derivation of pluripotent stem cells from both neonatal and adult mouse testis [28–30]. In these mouse studies, pluripotent stem cells were derived without genetic modification via enrichment for spermatogonial stem cells subsequent to reprogramming of MGSC colonies in culture. The resulting cells were shown to differentiate extensively to all germ layers and the germline, bearing resemblance to mouse embryonic stem cells [28–30].

In this study, we isolated pluripotent cells from human testis biopsies that were manually transferred onto MEFs and cultured under hESC culture conditions. On MEFs, the cells maintained the ability to form colonies for at least 20 passages. The colonies were characterized by a classic stem cell-like morphology on plates and the formation of EB-like structures, in suspension. On the basis of the results described above, we propose that these cells are hMGSCs.

### Diagnostic Gene Expression

The hMGSCs derived here expressed the pluripotency markers *OCT4* and *SOX-2,* but not *NANOG.* The apparent lack of NANOG expression is in agreement with the cell origin and NANOG expression pattern in adult testis in both humans and mice [22]. It is known that NANOG expression is regulated by the binding of OCT4 and SOX2 to the *NANOG* promoter region, and it has therefore been suggested that all three proteins function as regulators to maintain pluripotency [44]. Recent studies have demonstrated, however, that Nanog expression is not essential for self-renewal or the differentiation potential of embryonic stem cells but that instead it plays a role in establishing the inner cell mass and germ cells in vivo and that it enhances self-renewal of embryonic stem cells [44]. In addition to *OCT4* and *SOX2*, the expression of other genes, such as *STELLAR*, *GDF3*, *PUM1*, *PUM2, DAZL, SCP3*, and *MLH1*, was consistent with a germ cell origin of the hMGSCs [32, 45–47]. In contrast, expression of a late marker of male germ cell development (*TEKT1*) was not detected [48]. This expression pattern demonstrates that hMGSCs express pluripotency markers, early-stage germ cell markers, and a subset of later-stage germ cell markers. Furthermore, the distinct expression patterns of NK7 cells grown under ESC culture conditions and NK7 cells grown on human testicular stromal cells indicate that NK7 cells can be reprogrammed to hMGSCs, as demonstrated by *SOX2* expression and the loss of late germ cell marker expression.

### Spontaneous Differentiation In Vitro

The results from the spontaneous differentiation experiment clearly demonstrated that hMGSCs have the potential to differentiate into derivatives of the three primary germ layers in vitro. When transferred to differentiation media, the expression of *OCT4* decreased dramatically in hMGSCs, as well as in hESCs, in parallel. We also noted that the expression of *NCAM*, which has been used as an ectodermal marker for hESC differentiation, decreased during in vitro differentiation of hMGSCs. Although this was initially unexpected, closer scrutiny indicated that NCAM is expressed in male germline cells and may function in spermatogonial stem cells as a receptor for GDNF, as described [49, 50]. Thus, the decrease in *NCAM* expression further supports the results of somatic differentiation, as indicated by the increase in expression of the somatic differentiation markers *MSI1*, *GATA4*, and *KDR* at both the RNA and protein levels. These markers have previously been shown to be expressed solely in differentiated hESCs [51]. The differentiation potential of NK7 hMGSCs was further demonstrated by induced neural differentiation. Following 6 weeks of culture in neural cell-specific media, the expression of NES, MAP2, and TUB III could be detected.

### STR/VNTR, Karyotype, and Telomerase Activity

We observed that the karyotype of the putative hMGSCs, as well as the somatic cells of the patient who donated the biopsy, was normal. Furthermore, genetic analysis indicated that the hMGSCs were undoubtedly derived from the testis biopsy of the man who donated the sample for research and not a laboratory/cell contaminant. There was no evidence of common karyotypic abnormalities associated with germ cell tumors, such as amplification of chromosome 12p [52–55].

In addition to normal karyotype, hMGSCs possess telomerase activity in vitro. In immortal cells, such as hESCs, germ cells, or cancer cells, the shortening of telomere length is prevented by telomerase [56–58]. In this study, we observed a decrease in telomerase activity after two consecutive passages that may indicate that the current culture conditions require further optimization to enhance proliferative capacity, or stability, of the spermatogonial stem cells in vitro. Other cell types, including somatic cells and sperm, demonstrated little or no telomerase activity, as expected. These findings parallel those in mice [24, 28–30].

### Methylation Analysis

To further probe origins and status of the hMGSCs, we examined methylation of the imprinted locus, *H19*, a locus normally expressed differentially from the male and female germline. Numerous studies over the years have demonstrated that *H19* is methylated in the male germline; nonetheless, the timing of imprint erasure and the re-establishment of the male-specific methylation pattern in human germ cell development has not been completely elucidated. It seems most likely that de novo methylation is established before the germ cells enter meiosis [33]. Results of methylation analysis described above showed a ratio of 70% unmethylated to 30% methylated in hESCs, in line with previous studies of hESC imprints [12], and 50%:50% in human blood cells, as expected [38, 39]. Moreover, sperm cells carry only the paternal allele of the *H19* gene and were 100% methylated, which is also in agreement with published findings [33]. The hMGSCs at passage 8, however, were hypomethylated, with 87% of the clones being unmethylated and only 13% methylated, suggesting that either a subpopulation of germ cells (such as PGCs), devoid of methylation, gave rise to the hMGSCs or, alternatively, that reprogramming of the hMGSCs led to imprint erasure.

Recent studies have shown that reprogramming of somatic cells is associated with demethylation of *OCT4* regulatory regions, with the most apparent changes occurring in the PE, DE, and PP regions. Mosaic CpG demethylation has been shown to be physiologically important, as it leads to the activation of the gene. Analysis of the methylation status of the *OCT4* promoter region of NK7 cells at passages 2 and 8 demonstrated that 36% and 32% of CpG repeats are unmethylated. This partial demethylation is in agreement with the activation of the *OCT4* gene and supports the theory that human spermatogonial stem cells are multipotent when cultured under human ESC culture conditions.

### Teratoma Assay

The results of in vivo differentiation analysis merit further comment. We observed that hMGSCs did not induce formation of a large teratoma (which may or may not be beneficial for putative clinical applications). Nonetheless, PCR analysis using primers specific for the human *SRY* gene indicated the presence of human cells in the graft even after 2 months. The most likely explanation for this finding is that some of the human cells persist but that a larger number of cells is required for further teratoma analysis (4,000, as used here, is at the lower limit of detection without MEFs serving as a carrier [59]). Repetition of the teratoma assay using 10,000 hMGSCs accompanied by 1 million irradiated MEF cells did not lead to teratoma formation after transplantation, even though 500–1,000 murine embryonic stem cells accompanied by 99,000 MEFs have been shown to be sufficient to induce tumor growth [59]. These results indicate that although hMGSCs appear to have the potential to differentiate into derivatives of the three germ layers upon spontaneous or induced in vitro differentiation, they may not have been reprogrammed sufficiently to generate teratomas.

## CONCLUSION

The ability to isolate and culture hMGSCs in vitro may facilitate development of novel therapeutic strategies for the treatment of infertility. For example, one side effect of cancer treatments is the potential destruction of spermatogonial stem cells, along with the cancer cells, with the possibility of leaving the patient infertile [60]. To maintain fertility, testicular biopsies could be obtained prior to the treatment, and spermatogonial stem cells could be propagated in vitro and finally transplanted back into the patient's testis when the treatment is completed, if germ cell development can be controlled.

Recent studies in mice have also indicated that spermatogonial stem cells can acquire ESC traits via an as yet poorly defined reprogramming process [28–30]. We anticipate that pluripotent hMGSCs represent a source of patient-specific stem cells appropriate for the study of genetic diseases in different cell lineages in vitro and for potential novel therapeutic applications with particular application to fertility [61]. Notably, these cells are not genetically modified as is required for generation of induced pluripotent stem cells that can be derived from fetal or adult somatic cells [41–43]. Nonetheless, our results suggest that the efficient derivation of hMGSCs may require full reprogramming through the optimization of the culture conditions. In this regard, recent studies in nonhuman primates illuminate fundamental properties of spermatogonia that may inform future efforts [62].
