# Supplementary figures and images for: Isolation and Characterization of Pluripotent Human Spermatogonial Stem Cell-Derived Cells
Source: Stem Cells. 2009 Jan;27(1):138–49. doi: 10.1634/stemcells.2008-0439 (PMC2729695; doi:10.1634/stemcells.2008-0439)

A

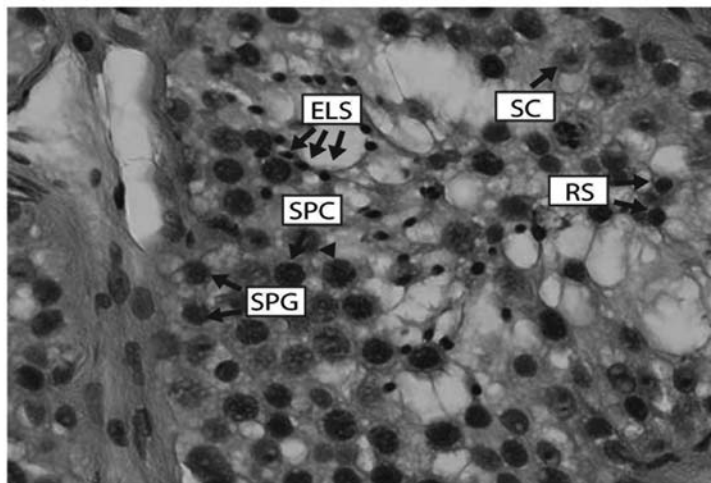

B

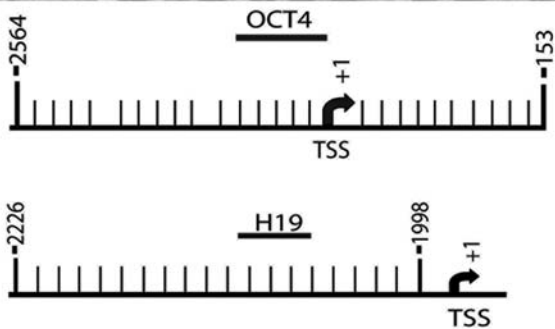

C

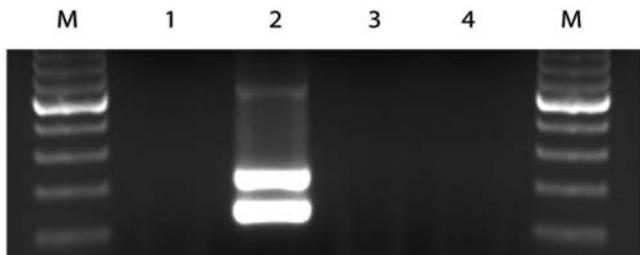

Supplement: Supplementary file 1 [file stem0027-0138-SD1.pdf]
